# Supplementary material for: Coping Mechanisms during the War in Ukraine: A Cross-Sectional Assessment among Romanian Population
Source: Healthcare (Basel). 2023 May 13;11(10):1412. doi: 10.3390/healthcare11101412 (PMC10218351; doi:10.3390/healthcare11101412)
Supplement: Supplementary file 1 [file healthcare-11-01412-s001.zip › Google Forms questionnaire.pdf]

Sănătatea mentală în vreme de război – un studiu longitudinal despre mecanisme de adaptare, anxietate și calitatea vieții

*Obligatoriu

Termeni si conditii

Vă invităm să participaţi la acest studiu clinic. Înainte de a decide este foarte important să înţelegeţi scopul acestui studiu şi ce implică acesta. Aceasta secțiune reprezintă o fişă de informare care vă va oferi detalii despre proiect. Vă rugăm să vă rezervaţi un minut pentru a citi cu atenţie următoarele informaţii. Dacă există nelămuriri sau dacă aveţi nevoie de mai multe informaţii vă stăm la dispoziţie.

Care este scopul acestui studiu?
Scopul lucrării constă în evaluarea mecanismelor de adaptare, a calității vieții și a nivelului de anxietate în rândul populației generale adulte, din perioada conflictului militar demarat in 24.02.2022 între Rusia și Ucraina, precum și în perioada imediat următoare, dacă acesta va lua sfârșit. Studiul se va desfășura pe o perioadă de 2 luni.

În cadrul Disciplinei de Psihiatrie și Psihiatrie Pediatrică Cluj-Napoca a Universității de Medicină și Farmacie "Iuliu Hațieganu" Cluj-Napoca se desfăşoară un studiu ce își propune evaluarea calității vieții, nivelului de anxietate si a mecanismelor de adaptare în rândul populației generale, in timpul conflictului militar desfășurat începând cu 24.02.2022 in Ucraina, cât și după încheierea acestuia, ținând cont de varietatea mare de stresori atât din viața personală cât și din cea profesională, dar și de reglementările legislative, posibilitatea extinderii conflictului spre România si consecințele economice ale conflictului militar.

Trebuie sa particip?
Participarea dumneavoastră la acest studiu este absolut voluntară. Este decizia Dumneavoastră dacă doriţi sau nu să luaţi parte la acest studiu. În orice moment vă puteti retrage din acest studiu. Dacă sunteţi de acord să participaţi, atunci vă vom solicita exprimarea consintământului informat, bifând DA la secțiunea Termeni și Condiții de la finalul acestei informări. De asemenea, acest studiu se adresează persoanelor care au împlinit vârsta de 18 ani. Studiul a primit avizul Comisiei de etică din cadrul UMF Iuliu Hațieganu Cluj-Napoca.

Ce am de făcut?
Participarea Dumneavoastră la acest studiu implică completarea unui chestionar, lucru ce va dura aproximativ 30 minute.

Prelucrarea datelor cu caracter personal (declaratie GDPR)
Rezultatele acestui studiu de cercetare pot fi prezentate la manifestări științifice sau medicale sau publicate în reviste științifice. Identitatea și /sau informațiile dumneavoastră personale nu vor fi divulgate, cu excepția celor autorizate de dvs. sau în conformitate cu legea.

Conform cerinţelor Regulamentul (UE) 2016/679 privind protecţia persoanelor fizice în ceea ce priveşte prelucrarea datelor cu caracter personal şi privind libera circulaţie a acestor date şi de abrogare a Directivei 95/46/CE (Regulamentul general privind protecţia datelor) şi ale Legii nr. 506/2004 privind prelucrarea datelor cu caracter personal şi protecţia vieţii private, echipa de cercetare are obligaţia de a administra în condiţii de siguranţă şi numai pentru scopurile specificate, datele pe care le veţi furniza: date socio-demografice şi răspunsuri subiective la chestionare.

Pe durata desfășurării studiului, datele vor fi colectate și stocate online pe platforma Google Drive. Această platformă a întreprins acțiunile necesare pentru a se alinia cerințelor Regulamentului (UE) 2016/679 privind protecţia persoanelor fizice în ceea ce priveşte prelucrarea datelor cu caracter personal şi privind libera circulaţie a acestor date şi de abrogare a Directivei 95/46/CE (Regulamentul general privind protecţia datelor).

1. Sunt de acord cu termenii si conditiile

Marcați un singur oval.

- [ ] DA *Treceți la întrebarea 2*
- [ ] NU *Treceți la secțiunea 6 (Va multumim pentru participare!).*

Date Generale

I. Date generale

2. Sex *

Bifați toate variantele aplicabile.

- [ ] Masculin
- [ ] Feminin

3. Vârstă (ani) *

Bifați toate variantele aplicabile.

- [ ] 18-25
- [ ] 26-30
- [ ] 31-40
- [ ] 41-50
- [ ] 51-60
- [ ] 61-70
- [ ] 71+

4. Ultima formă de educație absolvită

Bifați toate variantele aplicabile.

- [ ] Liceu
- [ ] Facultate
- [ ] Studii post-universitare

Altele: [ ] ______________________

5. Domiciliu *

Bifați toate variantele aplicabile.

- [ ] Urban
- [ ] Rural

6. Status Marital *

Bifați toate variantele aplicabile.

- [ ] Necăsătorit/ă
- [ ] Căsătorit/ă
- [ ] Divorțat/ă
- [ ] Văduv/ă
- [ ] Concubinaj
- [ ] Separat
- [ ] Relație la distanță

Altele: [ ] ____________________

7. Status profesional *

Bifați toate variantele aplicabile.

- [ ] Angajat
- [ ] Șomer
- [ ] Antreprenor
- [ ] Liber profesionist
- [ ] Pensionar

Altele: [ ] ____________________

Informatii auxiliare

Următoarele întrebări sunt adresate pentru evaluarea comportamentelor cu potențial la risc și a eventualelor consulturi psihiatrice efectuate pe perioada conflictului armat

8. Cum apreciați consumul de tutun pe perioada conflictului armat: *

Bifați toate variantele aplicabile.

- [ ] Am început consumul
- [ ] Am crescut consumul de țigarete
- [ ] Am redus consumul de țigarete
- [ ] Am sistat consumul de țigarete
- [ ] Nu fumez

9. Pe perioada conflictului armat am consumat: *

Bifați toate variantele aplicabile.

- [ ] Nu consum alcool
- [ ] Nu mai mult de 6 pahare (150ml/pahar) de vin săptămânal sau echivalent alcool
- [ ] Între 6-12 pahare (150ml/pahar) de vin săptămânal sau echivalent alcool
- [ ] Mai mult de 12 pahare (150ml/pahar) de vin săptămânal sau echivalent alcool
- [ ] Mai mult de 12 pahare (150ml/pahar) de vin zilnic sau echivalent alcool

Echivalente consum alcool

1 pahar vin 150ml 14% = 1 pahar bere 500ml 5% = 1 shot tărie 50ml 40%

10. Pe perioada conflictului armat am consumat alte substanțe psihoactive față de cele menționate anterior: *

Bifați toate variantele aplicabile.

- [ ] Da
- [ ] Nu

11. Pe perioada conflictului armat am apelat la un consult de specialitate pentru: *

Bifați toate variantele aplicabile.

- [ ] Nu am apelat
- [ ] Stări anxioase
- [ ] Stări depresive
- [ ] Probleme cu consumul de substanțe
- [ ] Tulburări de somn

Altele: [ ] ______________________

12. În cazul în care vă cunoașteți cu o patologie psihiatrică, ați avut un episod de recădere pe perioada conflictului armat? *

Bifați toate variantele aplicabile.

- [ ] Da
- [ ] Nu

Chestionarului WHO-QOL-BREF

III. Aplicarea chestionarului WHO-QOL-BREF

Acest chestionar vă întreabă despre cum simțiți calitatea vieții Dvs, sănătatea și alte aspecte ale vieții. Vă rugăm să răspundeți la toate întrebările raportat la perioada conflictului armat

13. 1(G1) Cum ați aprecia calitatea vieții dumneavoastră? *

Bifați toate variantele aplicabile.

- [ ] Foarte prost
- [ ] Prost
- [ ] Nici prost nici bine
- [ ] Bine
- [ ] Foarte bine

14. 2(G4) Cât de satisfacut sunteți de sănătatea Dvs? *

Bifați toate variantele aplicabile.

- [ ] Foarte nesatisfăcut
- [ ] Nesatisfăcut
- [ ] Nici satisfăcut nici nestisfăcut
- [ ] Satisfăcut
- [ ] Foarte satisfăcut

Următoarele întrebări sunt despre cât de mult ați trăit anumite lucruri în perioada conflictului armat

15. 3(F1.4) Cât de tare ați simtit că durerea v-a împiedicat să faceți ceea ce era nevoie să faceți? *

Bifați toate variantele aplicabile.

- [ ] Deloc
- [ ] Puțin
- [ ] Moderat
- [ ] Foarte mult
- [ ] Extrem de mult

16. 4(F11.3) Cât de mult aveți nevoie de vreun tratament medical ca să vă duceți viața de zi cu zi? *

Bifați toate variantele aplicabile.

- [ ] Deloc
- [ ] Puțin
- [ ] Moderat
- [ ] Foarte mult
- [ ] Extrem de mult

17. 5(F4.1) Cât de mult vă bucurați de viață? *

Bifați toate variantele aplicabile.

- [ ] Deloc
- [ ] Puțin
- [ ] Moderat
- [ ] Foarte mult
- [ ] Extrem de mult

18. 6(F24.2) Cât de mult simțiți că viața Dvs este importantă *

Bifați toate variantele aplicabile.

- [ ] Deloc
- [ ] Puțin
- [ ] Moderat
- [ ] Foarte mult
- [ ] Extrem de mult

19. 7(F5.3) Cât de bine vă puteți concentra? *

Bifați toate variantele aplicabile.

- [ ] Deloc
- [ ] Puțin
- [ ] Moderat
- [ ] Foarte mult
- [ ] Extrem de mult

20. 8(F16.1) Cât de sigur vă simțiți în viața de zi cu zi? *

Bifați toate variantele aplicabile.

- [ ] Deloc
- [ ] Puțin
- [ ] Moderat
- [ ] Foarte mult
- [ ] Extrem de mult

21. 9(F22.1) Cât de sănătoasă este ambianța Dvs fizică? *

Bifați toate variantele aplicabile.

- [ ] Deloc
- [ ] Puțin
- [ ] Moderat
- [ ] Foarte mult
- [ ] Extrem de mult

Următoarele întrebări sunt despre cât de intens ați trăit unele lucruri în perioada conflictului armat

22. 10(F2.1) Ați avut suficientă energie pentru viața de zi cu zi? *

Bifați toate variantele aplicabile.

- [ ] Deloc
- [ ] Puțin
- [ ] Moderat
- [ ] Foarte mult
- [ ] Extrem de mult

23. 11(F7.1) Ați acceptat înfățișarea Dvs exterioară? *

Bifați toate variantele aplicabile.

- [ ] Deloc
- [ ] Puțin
- [ ] Moderat
- [ ] Foarte mult
- [ ] Extrem de mult

24. 12(F18.1) Ați avut suficienți bani pentru nevoile Dvs? *

Bifați toate variantele aplicabile.

- [ ] Deloc
- [ ] Puțin
- [ ] Moderat
- [ ] Foarte mult
- [ ] Extrem de mult

25. 13(F20.1) Cât de accesibilă este informația de care aveți nevoie în viața de zi cu zi? *

Bifați toate variantele aplicabile.

- [ ] Deloc
- [ ] Puțin
- [ ] Moderat
- [ ] Foarte mult
- [ ] Extrem de mult

26. 14(F21.1) Cât de tare ați avut ocazia să vă recreați? *

Bifați toate variantele aplicabile.

- [ ] Deloc
- [ ] Puțin
- [ ] Moderat
- [ ] Foarte mult
- [ ] Extrem de mult

27. 15(F9.1) Cât de bine ați putut să vă deplasați? *

Bifați toate variantele aplicabile.

- [ ] Deloc
- [ ] Puțin
- [ ] Moderat
- [ ] Foarte mult
- [ ] Extrem de mult

Următoarele întrebări sunt despre cât de bine sau satisfăcut ați fost de variatele aspecte ale vieții Dvs în perioada conflictului armat

28. 16(F3.3) Cât de satisfăcut sunteți de somnul Dvs? *

Bifați toate variantele aplicabile.

- [ ] Foarte nesatisfăcut
- [ ] Nesatisfăcut
- [ ] Nici satisfăcut nici nesatisfăcut
- [ ] Satisfăcut
- [ ] Foarte satisfăcut

29. 17(F10.3) Cât de satisfăcut sunteți de capacitatea Dvs de a îndeplini activitățile vieții de zi cu zi? *

Bifați toate variantele aplicabile.

- [ ] Foarte nesatisfăcut
- [ ] Nesatisfăcut
- [ ] Nici satisfăcut nici nesatisfăcut
- [ ] Satisfăcut
- [ ] Foarte satisfăcut

30. 18(F12.4) Cât de satisfăcut sunteți de randamentul Dvs de muncă? *

Bifați toate variantele aplicabile.

- [ ] Foarte nesatisfăcut
- [ ] Nesatisfăcut
- [ ] Nici satisfăcut nici nesatisfăcut
- [ ] Satisfăcut
- [ ] Foarte satisfăcut

31. 19(F6.3) Cât de satisfăcut sunteți de Dvs înșivă? *

Bifați toate variantele aplicabile.

- [ ] Foarte nesatisfăcut
- [ ] Nesatisfăcut
- [ ] Nici satisfăcut nici nesatisfăcut
- [ ] Satisfăcut
- [ ] Foarte satisfăcut

32. 20(F13.3) Cât de satisfăcut sunteți de relațiile Dvs personale? *

Bifați toate variantele aplicabile.

- [ ] Foarte nesatisfăcut
- [ ] Nesatisfăcut
- [ ] Nici satisfăcut nici nesatisfăcut
- [ ] Satisfăcut
- [ ] Foarte satisfăcut

33. 21(F15.3) Cât de satisfăcut sunteți de viața sexuală a Dvs? *

Bifați toate variantele aplicabile.

- [ ] Foarte nesatisfăcut
- [ ] Nesatisfăcut
- [ ] Nici satisfăcut nici nesatisfăcut
- [ ] Satisfăcut
- [ ] Foarte satisfăcut

34. 22(F14.4) Cât de satisfăcut sunteți de sprijinul pe care-l aveți de la prietenii Dvs? *

Bifați toate variantele aplicabile.

- [ ] Foarte nesatisfăcut
- [ ] Nesatisfăcut
- [ ] Nici satisfăcut nici nesatisfăcut
- [ ] Satisfăcut
- [ ] Foarte satisfăcut

35. 23(F17.3) Cât de statisfăcut sunteți de locul unde locuiți? *

Bifați toate variantele aplicabile.

- [ ] Foarte nesatisfăcut
- [ ] Nesatisfăcut
- [ ] Nici satisfăcut nici nesatisfăcut
- [ ] Satisfăcut
- [ ] Foarte satisfăcut

36. 24(F19.3) Cât de satisfăcut sunteți de accesibilitatea la serviciile de sănătate? *

Bifați toate variantele aplicabile.

- [ ] Foarte nesatisfăcut
- [ ] Nesatisfăcut
- [ ] Nici satisfăcut nici nesatisfăcut
- [ ] Satisfăcut
- [ ] Foarte satisfăcut

37. 25(F23.3) Cât de satisfăcut sunteți de modul Dvs de transport? *

Bifați toate variantele aplicabile.

- [ ] Foarte nesatisfăcut
- [ ] Nesatisfăcut
- [ ] Nici satisfăcut nici nesatisfăcut
- [ ] Satisfăcut
- [ ] Foarte satisfăcut

Următoarea întrebare se referă la cât de des ați simțit sau trăit anumite lucruri în perioada conflictului armat

38. 26(F8.1) Cât de des ați trăit sentimente negative precum tristețe, disperare, teamă, nefericire? *

Bifați toate variantele aplicabile.

- [ ] Niciodată
- [ ] Rareori
- [ ] Aproape des
- [ ] Foarte des
- [ ] Întotdeauna

HRSA - Scala de anxietate Hamilton

Anxietatea este un termen care semnifică modificări specifice la patru nivele. Astfel, persoanele cu anxietate experimentează sentimente de teamă, catastrofă iminentă, neputinţă, groază (nivelul subiectiv). HARS este utilizată pentru a evalua severitatea simptomelor anxietăţii, atât la copii cât şi la adulţi. HARS a fost concepută în 1959 de către Max Hamilton şi este unul dintre primele instrumente elaborate pentru a cuantifica severitatea simptomatologiei de tip anxios. Scala permite o evaluare globală a simptomelor psihice (ex., tensiune psihică, dispoziţie anxioasă) şi somatice (ex., modificări bio-fiziologice) ale anxietăţii.

*** Va rugam sa raspundeti despre cum va simtiti strict pe perioada conflictului militar

39. 1. Dispoziţie anxioasă – nelinişte, anticipare a ceea ce este mai rău, anticiparea producerii unui lucru îngrozitor, iritabilitate. *

Marcați un singur oval.

- Absent
- Ușor
- Moderat
- Sever
- Foarte sever

40. 2. Tensiune – sentiment de tensiune (încordare), oboseală, reacţii de tresărire, tendinţa de a plânge foarte uşor, tremurături, sentimentul neliniştii, incapacitate de a se relaxa. *

Marcați un singur oval.

- Absent
- Ușor
- Moderat
- Sever
- Foarte sever

41. 3. Fobii – de întuneric, de persoane străine, de a fi lăsat singur, de animale, de trafic, de aglomeraţie. *

Marcați un singur oval.

- Absent
- Ușor
- Moderat
- Sever
- Foarte sever

42. 4. Insomnii – dificultăţi de adormire, somn întrerupt, somn neîndestulător şi oboseală la trezire, vise, coşmaruri, spaime nocturne. *

Marcați un singur oval.

- Absent
- Ușor
- Moderat
- Sever
- Foarte sever

43. 5. Deficienţe de concentrare – dificultăţi de concentrare, memorie slabă. *

Marcați un singur oval.

- Absent
- Ușor
- Moderat
- Sever
- Foarte sever

44. 6. Dispoziţie depresivă – lipsă de interes, lipsa de plăcere în hobby-uri, depresie, trezire matinală, agitaţie în timpul zilei. *

Marcați un singur oval.

- Absent
- Ușor
- Moderat
- Sever
- Foarte sever

45. 7. Simptome somatice musculare – dureri, contracţii musculare, scrâşnirea dinţilor, voce nesigură, tonus muscular crescut. *

Marcați un singur oval.

- Absent
- Ușor
- Moderat
- Sever
- Foarte sever

46. 8. Simptome somatice senzoriale – ţiuit în urechi, vedere înceţoşată, transpiraţii reci şi calde, sentiment de slăbiciune, senzaţia unor înţepături. *

Marcați un singur oval.

- Absent
- Ușor
- Moderat
- Sever
- Foarte sever

47. 9. Simptome cardiovasculare – tahicardie, palpitaţii, dureri în piept, senzaţii de leşin, oftat, dispnee (respiraţie grea) *

Marcați un singur oval.

- Absent
- Ușor
- Moderat
- Sever
- Foarte sever

48. 10. Simptome respiratorii – presiune şi apăsare în piept, senzaţie de sufocare, oftat, dispnee. *

Marcați un singur oval.

- Absent
- Ușor
- Moderat
- Sever
- Foarte sever

49. 11. Simptome gastrointestinale – dificultăţi de înghiţire, dureri abdominale, senzaţie de arsuri la stomac, senzaţii de stomac plin, greaţă, vărsături, pierdere în greutate, diaree, constipaţie. *

Marcați un singur oval.

- Absent
- Ușor
- Moderat
- Sever
- Foarte sever

50. 12. Simptome genito-urinare – urinări frecvente, urgenţă la urinare, amenoree, frigiditate, ejaculare precoce, pierderea libidoului, impotenţă. *

Marcați un singur oval.

- Absent
- Ușor
- Moderat
- Sever
- Foarte sever

51. 13. Simptome vegetative – gură uscată, îmbujorări, paloare, transpiraţie, ameţeli, durere de cap intensă. *

Marcați un singur oval.

- Absent
- Ușor
- Moderat
- Sever
- Foarte sever

52. 14. Comportament – agitaţie, nelinişte, tremurul mâinilor, frunte încreţită, faţă încordată, suspine sau respiraţie rapidă, înghiţituri. *

Marcați un singur oval.

- Absent
- Ușor
- Moderat
- Sever
- Foarte sever

Scala COPE

Scala COPE

Ne interesează cum reacţionează oamenii când se confruntă cu evenimente dificile şi stresante în viaţă. Există multe modalităţi de a încerca să faci faţă stresului. Acest chestionar vă solicită să indicaţi ce faceţi şi simţiţi, în general, când trăiţi experienţa unor evenimente stresante. Evident, diferitele evenimente duc la reacţii diferite, dar gândiţi-vă la ce faceţi de obicei când sunteţi sub stres deosebit. Apoi răspundeţi la fiecare din itemii următori, bifând o căsuță pe formularul de răspuns pentru fiecare, folosind posibilităţile de alegere enunţate mai jos. Alegeţi-vă răspunsurile cu grijă. Nu există răspunsuri ,,corecte" sau"greşite", aşa că alegeţi cel mai potrivit răspuns pentru dv. Indicaţi ce faceţi dv. de obicei când trăiţi experienţa unui eveniment stresant, având următoarele modalităţi de răspuns:

1. De obicei nu fac asta deloc.
2. De obicei fac asta în mică masură.
3. De obicei fac asta în măsură medie.
4. De obicei fac asta în mare măsură.

53. 01. Încerc să mă dezvolt ca persoană ca rezultat al experienţei *

Bifați toate variantele aplicabile.

- [ ] De obicei nu fac asta deloc.
- [ ] De obicei fac asta în mică masură.
- [ ] De obicei fac asta în măsură medie.
- [ ] De obicei fac asta în mare măsură.

54. 02. Mă apuc de lucru sau de alte activităţi pentru a-mi lua gândurile de la anumite lucruri *

Bifați toate variantele aplicabile.

- [ ] De obicei nu fac asta deloc.
- [ ] De obicei fac asta în mică masură.
- [ ] De obicei fac asta în măsură medie.
- [ ] De obicei fac asta în mare măsură.

55. 03. Mă supăr şi-mi dau frâu liber emoţiilor *

Bifați toate variantele aplicabile.

- [ ] De obicei nu fac asta deloc.
- [ ] De obicei fac asta în mică masură.
- [ ] De obicei fac asta în măsură medie.
- [ ] De obicei fac asta în mare măsură.

56. 04. Încerc să obţin sfaturi de la cineva în legătură cu ceea ce trebuie să fac *

Bifați toate variantele aplicabile.

- [ ] De obicei nu fac asta deloc.
- [ ] De obicei fac asta în mică masură.
- [ ] De obicei fac asta în măsură medie.
- [ ] De obicei fac asta în mare măsură.

57. 05. Îmi concentrez eforturile să întreprind ceva în legătură cu situaţia *

Bifați toate variantele aplicabile.

- [ ] De obicei nu fac asta deloc.
- [ ] De obicei fac asta în mică masură.
- [ ] De obicei fac asta în măsură medie.
- [ ] De obicei fac asta în mare măsură.

58. 06. Îmi zic: ,,nu-i adevărat!" *

Bifați toate variantele aplicabile.

- [ ] De obicei nu fac asta deloc.
- [ ] De obicei fac asta în mică masură.
- [ ] De obicei fac asta în măsură medie.
- [ ] De obicei fac asta în mare măsură.

59. 07. Îmi plasez încrederea în Dumnezeu *

Bifați toate variantele aplicabile.

- [ ] De obicei nu fac asta deloc.
- [ ] De obicei fac asta în mică masură.
- [ ] De obicei fac asta în măsură medie.
- [ ] De obicei fac asta în mare măsură.

60. 08. Râd de situaţia respectivă *

Bifați toate variantele aplicabile.

- [ ] De obicei nu fac asta deloc.
- [ ] De obicei fac asta în mică masură.
- [ ] De obicei fac asta în măsură medie.
- [ ] De obicei fac asta în mare măsură.

61. 09. Recunosc în mine că nu pot face faţă şi nu mai încerc *

Bifați toate variantele aplicabile.

- [ ] De obicei nu fac asta deloc.
- [ ] De obicei fac asta în mică masură.
- [ ] De obicei fac asta în măsură medie.
- [ ] De obicei fac asta în mare măsură.

62. 10. Mă abţin de la a face ceva prea repede *

Bifați toate variantele aplicabile.

- [ ] De obicei nu fac asta deloc.
- [ ] De obicei fac asta în mică masură.
- [ ] De obicei fac asta în măsură medie.
- [ ] De obicei fac asta în mare măsură.

63. 11. Discut ceea ce simt cu cineva *

Bifați toate variantele aplicabile.

- [ ] De obicei nu fac asta deloc.
- [ ] De obicei fac asta în mică masură.
- [ ] De obicei fac asta în măsură medie.
- [ ] De obicei fac asta în mare măsură.

64. 12. Folosesc alcool sau droguri pentru a mă simţi mai bine *

Bifați toate variantele aplicabile.

- [ ] De obicei nu fac asta deloc.
- [ ] De obicei fac asta în mică masură.
- [ ] De obicei fac asta în măsură medie.
- [ ] De obicei fac asta în mare măsură.

65. 13. Mă obişnuiesc cu ideea că s-a întâmplat *

Bifați toate variantele aplicabile.

- [ ] De obicei nu fac asta deloc.
- [ ] De obicei fac asta în mică masură.
- [ ] De obicei fac asta în măsură medie.
- [ ] De obicei fac asta în mare măsură.

66. 14. Discut cu cineva pentru a afla lucruri în plus despre situaţie *

Bifați toate variantele aplicabile.

- [ ] De obicei nu fac asta deloc.
- [ ] De obicei fac asta în mică masură.
- [ ] De obicei fac asta în măsură medie.
- [ ] De obicei fac asta în mare măsură.

67. 15. Mă feresc să fiu distras de alte gânduri sau activităţi *

Bifați toate variantele aplicabile.

- [ ] De obicei nu fac asta deloc.
- [ ] De obicei fac asta în mică masură.
- [ ] De obicei fac asta în măsură medie.
- [ ] De obicei fac asta în mare măsură.

68. 16. Visez cu ochii deschişi la alte lucruri *

Bifați toate variantele aplicabile.

- [ ] De obicei nu fac asta deloc.
- [ ] De obicei fac asta în mică masură.
- [ ] De obicei fac asta în măsură medie.
- [ ] De obicei fac asta în mare măsură.

69. 17. Mă supăr şi sunt realmente conştient de asta *

Bifați toate variantele aplicabile.

- [ ] De obicei nu fac asta deloc.
- [ ] De obicei fac asta în mică masură.
- [ ] De obicei fac asta în măsură medie.
- [ ] De obicei fac asta în mare măsură.

70. 18. Caut ajutor la Dumnezeu *

Bifați toate variantele aplicabile.

- [ ] De obicei nu fac asta deloc.
- [ ] De obicei fac asta în mică masură.
- [ ] De obicei fac asta în măsură medie.
- [ ] De obicei fac asta în mare măsură.

71. 19. Îmi fac un plan de acţiune *

Bifați toate variantele aplicabile.

- [ ] De obicei nu fac asta deloc.
- [ ] De obicei fac asta în mică masură.
- [ ] De obicei fac asta în măsură medie.
- [ ] De obicei fac asta în mare măsură.

72. 20. Glumesc pe seama situaţiei *

Bifați toate variantele aplicabile.

- [ ] De obicei nu fac asta deloc.
- [ ] De obicei fac asta în mică masură.
- [ ] De obicei fac asta în măsură medie.
- [ ] De obicei fac asta în mare măsură.

73. 21. Accept că s-a întâmplat şi că nu se poate schimba nimic *

Bifați toate variantele aplicabile.

- [ ] De obicei nu fac asta deloc.
- [ ] De obicei fac asta în mică masură.
- [ ] De obicei fac asta în măsură medie.
- [ ] De obicei fac asta în mare măsură.

74. 22. Amân a face ceva în legătură cu problema până situaţia o permite *

Bifați toate variantele aplicabile.

- [ ] De obicei nu fac asta deloc.
- [ ] De obicei fac asta în mică masură.
- [ ] De obicei fac asta în măsură medie.
- [ ] De obicei fac asta în mare măsură.

75. 23. Încerc să obţin sprijin emoţional de la prieteni sau rude *

Bifați toate variantele aplicabile.

- [ ] De obicei nu fac asta deloc.
- [ ] De obicei fac asta în mică masură.
- [ ] De obicei fac asta în măsură medie.
- [ ] De obicei fac asta în mare măsură.

76. 24. Pur şi simplu renunţ la atingerea scopului *

Bifați toate variantele aplicabile.

- [ ] De obicei nu fac asta deloc.
- [ ] De obicei fac asta în mică masură.
- [ ] De obicei fac asta în măsură medie.
- [ ] De obicei fac asta în mare măsură.

77. 25. Încerc acţiuni suplimentare pentru a scăpa de problemă *

Bifați toate variantele aplicabile.

- [ ] De obicei nu fac asta deloc.
- [ ] De obicei fac asta în mică masură.
- [ ] De obicei fac asta în măsură medie.
- [ ] De obicei fac asta în mare măsură.

78. 26. Încerc să uit de mine pentru un timp consumând alcool sau luând droguri *

Bifați toate variantele aplicabile.

- [ ] De obicei nu fac asta deloc.
- [ ] De obicei fac asta în mică masură.
- [ ] De obicei fac asta în măsură medie.
- [ ] De obicei fac asta în mare măsură.

79. 27. Refuz să cred că s-a întâmplat *

Bifați toate variantele aplicabile.

- [ ] De obicei nu fac asta deloc.
- [ ] De obicei fac asta în mică masură.
- [ ] De obicei fac asta în măsură medie.
- [ ] De obicei fac asta în mare măsură.

80. 28. Îmi dau frâu liber simţămintelor *

Bifați toate variantele aplicabile.

- [ ] De obicei nu fac asta deloc.
- [ ] De obicei fac asta în mică masură.
- [ ] De obicei fac asta în măsură medie.
- [ ] De obicei fac asta în mare măsură.

81. 29. Încerc să văd problema în lumină diferită, pentru a o face să pară mai pozitivă *

Bifați toate variantele aplicabile.

- [ ] De obicei nu fac asta deloc.
- [ ] De obicei fac asta în mică masură.
- [ ] De obicei fac asta în măsură medie.
- [ ] De obicei fac asta în mare măsură.

82. 30. Vorbesc cu cineva care ar putea face ceva concret în legătură cu problema *

Bifați toate variantele aplicabile.

- [ ] De obicei nu fac asta deloc.
- [ ] De obicei fac asta în mică masură.
- [ ] De obicei fac asta în măsură medie.
- [ ] De obicei fac asta în mare măsură.

83. 31. Dorm mai mult ca de obicei *

Bifați toate variantele aplicabile.

- [ ] De obicei nu fac asta deloc.
- [ ] De obicei fac asta în mică masură.
- [ ] De obicei fac asta în măsură medie.
- [ ] De obicei fac asta în mare măsură.

84. 32. Încerc sa-mi fac o strategie legată de ceea ce este de făcut *

Bifați toate variantele aplicabile.

- [ ] De obicei nu fac asta deloc.
- [ ] De obicei fac asta în mică masură.
- [ ] De obicei fac asta în măsură medie.
- [ ] De obicei fac asta în mare măsură.

85. 33. Mă concentrez pe abordarea problemei şi, dacă este necesar, las alte lucruri deoparte un timp *

Bifați toate variantele aplicabile.

- [ ] De obicei nu fac asta deloc.
- [ ] De obicei fac asta în mică masură.
- [ ] De obicei fac asta în măsură medie.
- [ ] De obicei fac asta în mare măsură.

86. 34. Obţin compasiune şi înţelegere de la cineva *

Bifați toate variantele aplicabile.

- [ ] De obicei nu fac asta deloc.
- [ ] De obicei fac asta în mică masură.
- [ ] De obicei fac asta în măsură medie.
- [ ] De obicei fac asta în mare măsură.

87. 35. Beau alcool sau iau droguri, pentru a mă gândi mai puţin la problemă *

Bifați toate variantele aplicabile.

- [ ] De obicei nu fac asta deloc.
- [ ] De obicei fac asta în mică masură.
- [ ] De obicei fac asta în măsură medie.
- [ ] De obicei fac asta în mare măsură.

88. 36. Glumesc despre problemă *

Bifați toate variantele aplicabile.

- [ ] De obicei nu fac asta deloc.
- [ ] De obicei fac asta în mică masură.
- [ ] De obicei fac asta în măsură medie.
- [ ] De obicei fac asta în mare măsură.

89. 37. Renunţ la încercarea de a obţine ce doresc *

Bifați toate variantele aplicabile.

- [ ] De obicei nu fac asta deloc.
- [ ] De obicei fac asta în mică masură.
- [ ] De obicei fac asta în măsură medie.
- [ ] De obicei fac asta în mare măsură.

90. 38. Caut ceva bun în ceea ce se întâmplă *

Bifați toate variantele aplicabile.

- [ ] De obicei nu fac asta deloc.
- [ ] De obicei fac asta în mică masură.
- [ ] De obicei fac asta în măsură medie.
- [ ] De obicei fac asta în mare măsură.

91. 39. Mă gândesc cum aş putea aborda problema cel mai bine *

Bifați toate variantele aplicabile.

- [ ] De obicei nu fac asta deloc.
- [ ] De obicei fac asta în mică masură.
- [ ] De obicei fac asta în măsură medie.
- [ ] De obicei fac asta în mare măsură.

92. 40. Mă prefac că nu s-a întâmplat în realitate *

Bifați toate variantele aplicabile.

- [ ] De obicei nu fac asta deloc.
- [ ] De obicei fac asta în mică masură.
- [ ] De obicei fac asta în măsură medie.
- [ ] De obicei fac asta în mare măsură.

93. 41. Mă asigur că nu fac problema mai dificilă acţionând prea repede *

Bifați toate variantele aplicabile.

- [ ] De obicei nu fac asta deloc.
- [ ] De obicei fac asta în mică masură.
- [ ] De obicei fac asta în măsură medie.
- [ ] De obicei fac asta în mare măsură.

94. 42. Încerc din răsputeri să nu las alte lucruri să interfere cu eforturile mele în timp ce mă ocup de problemă *

Bifați toate variantele aplicabile.

- [ ] De obicei nu fac asta deloc.
- [ ] De obicei fac asta în mică masură.
- [ ] De obicei fac asta în măsură medie.
- [ ] De obicei fac asta în mare măsură.

95. 43. Merg la film sau urmăresc programe TV, pentru a mă gândi mai puţin la problemă *

Bifați toate variantele aplicabile.

- [ ] De obicei nu fac asta deloc.
- [ ] De obicei fac asta în mică masură.
- [ ] De obicei fac asta în măsură medie.
- [ ] De obicei fac asta în mare măsură.

96. 44. Accept realitatea faptului că s-a întâmplat *

Bifați toate variantele aplicabile.

- [ ] De obicei nu fac asta deloc.
- [ ] De obicei fac asta în mică masură.
- [ ] De obicei fac asta în măsură medie.
- [ ] De obicei fac asta în mare măsură.

97. 45. Întreb oamenii care au avut experienţe similare ce au făcut *

Bifați toate variantele aplicabile.

- [ ] De obicei nu fac asta deloc.
- [ ] De obicei fac asta în mică masură.
- [ ] De obicei fac asta în măsură medie.
- [ ] De obicei fac asta în mare măsură.

98. 46. Simt mult disconfort emoţional şi mă trezesc exprimându-mi aceste sentimente în mare măsură *

Bifați toate variantele aplicabile.

- [ ] De obicei nu fac asta deloc.
- [ ] De obicei fac asta în mică masură.
- [ ] De obicei fac asta în măsură medie.
- [ ] De obicei fac asta în mare măsură.

99. 47. Acţionez direct pentru a controla problema *

Bifați toate variantele aplicabile.

- [ ] De obicei nu fac asta deloc.
- [ ] De obicei fac asta în mică masură.
- [ ] De obicei fac asta în măsură medie.
- [ ] De obicei fac asta în mare măsură.

100. 48. Îmi caut consolare în religia mea *

Bifați toate variantele aplicabile.

- [ ] De obicei nu fac asta deloc.
- [ ] De obicei fac asta în mică masură.
- [ ] De obicei fac asta în măsură medie.
- [ ] De obicei fac asta în mare măsură.

101. 49. Mă oblig să aştept momentul propice pentru a face ceva *

Bifați toate variantele aplicabile.

- [ ] De obicei nu fac asta deloc.
- [ ] De obicei fac asta în mică masură.
- [ ] De obicei fac asta în măsură medie.
- [ ] De obicei fac asta în mare măsură.

102. 50. Râd de situaţie *

Bifați toate variantele aplicabile.

- [ ] De obicei nu fac asta deloc.
- [ ] De obicei fac asta în mică masură.
- [ ] De obicei fac asta în măsură medie.
- [ ] De obicei fac asta în mare măsură.

103. 51. Reduc cantitatea de efort consacrată rezolvării problemei *

Bifați toate variantele aplicabile.

- [ ] De obicei nu fac asta deloc.
- [ ] De obicei fac asta în mică masură.
- [ ] De obicei fac asta în măsură medie.
- [ ] De obicei fac asta în mare măsură.

104. 52. Îi spun cuiva despre cum mă simt *

Bifați toate variantele aplicabile.

- [ ] De obicei nu fac asta deloc.
- [ ] De obicei fac asta în mică masură.
- [ ] De obicei fac asta în măsură medie.
- [ ] De obicei fac asta în mare măsură.

105. 53. Consum alcool sau droguri pentru a mă ajuta să trec prin situaţie *

Bifați toate variantele aplicabile.

- [ ] De obicei nu fac asta deloc.
- [ ] De obicei fac asta în mică masură.
- [ ] De obicei fac asta în măsură medie.
- [ ] De obicei fac asta în mare măsură.

106. 54. Învăţ să trăiesc cu situaţia *

Bifați toate variantele aplicabile.

- [ ] De obicei nu fac asta deloc.
- [ ] De obicei fac asta în mică masură.
- [ ] De obicei fac asta în măsură medie.
- [ ] De obicei fac asta în mare măsură.

107. 55. Las la o parte alte activităţi pentru a mă concentra asupra problemei *

Bifați toate variantele aplicabile.

- [ ] De obicei nu fac asta deloc.
- [ ] De obicei fac asta în mică masură.
- [ ] De obicei fac asta în măsură medie.
- [ ] De obicei fac asta în mare măsură.

108. 56. Mă gândesc mult la ce paşi să întreprind *

Bifați toate variantele aplicabile.

- [ ] De obicei nu fac asta deloc.
- [ ] De obicei fac asta în mică masură.
- [ ] De obicei fac asta în măsură medie.
- [ ] De obicei fac asta în mare măsură.

109. 57. Acţionez de parcă nu s-a întâmplat *

Bifați toate variantele aplicabile.

- [ ] De obicei nu fac asta deloc.
- [ ] De obicei fac asta în mică masură.
- [ ] De obicei fac asta în măsură medie.
- [ ] De obicei fac asta în mare măsură.

110. 58. Fac ceea ce e de făcut, pas cu pas *

Bifați toate variantele aplicabile.

- [ ] De obicei nu fac asta deloc.
- [ ] De obicei fac asta în mică masură.
- [ ] De obicei fac asta în măsură medie.
- [ ] De obicei fac asta în mare măsură.

111. 59. Învăţ ceva din experienţa asta *

Bifați toate variantele aplicabile.

- [ ] De obicei nu fac asta deloc.
- [ ] De obicei fac asta în mică masură.
- [ ] De obicei fac asta în măsură medie.
- [ ] De obicei fac asta în mare măsură.

112. 60. Mă rog mai mult ca de obicei *

Bifați toate variantele aplicabile.

- [ ] De obicei nu fac asta deloc.
- [ ] De obicei fac asta în mică masură.
- [ ] De obicei fac asta în măsură medie.
- [ ] De obicei fac asta în mare măsură.

Va multumim pentru participare!

Acest conținut nu este nici creat, nici aprobat de Google.

Formulare Google
