# Supplementary material for: Coping Mechanisms during the War in Ukraine: A Cross-Sectional Assessment among Romanian Population
Source: Healthcare (Basel). 2023 May 13;11(10):1412. doi: 10.3390/healthcare11101412 (PMC10218351; doi:10.3390/healthcare11101412)
Supplement: Supplementary file 1 [file healthcare-11-01412-s001.zip › Rstudio script for data analysis.pdf]

```
# packages

library(dplyr)
library(psych)
library(readxl)
library(bootnet)
library(qgraph)
library(rempsyc)
library(olsrr)
library(mice)


### import dataset

Data <- read_excel("Data1.xlsx") %>% as.data.frame()

#### descriptive demographics
Data$Sex %>% table()
Data$Age %>% table()
Data$education %>% table()
Data$Domiciliu%>% table()
Data$marital_status %>% table()
Data$professional_status %>% table()
### code variables


Data$WHOQOL_1 = recode(Data$WHOQOL_1,
                       'Foarte prost' =1,
                       'Prost' = 2,
                       'Nici prost nici bine' = 3,
                       'Bine' = 4,
                       'Foarte bine' = 5
)



Data$WHOQOL_2 = recode(Data$WHOQOL_2,
       'Foarte nesatisfacut' =1,
       'Nesatisfacut' = 2,
       'Nici satisfacut nici nestisfacut' = 3,
       'Satisfacut' = 4,
       'Foarte satisfacut' = 5
       )


for(i in 14:26) {
  Data[,i] =  Data[,i] %>% c() %>% unlist() %>% c() %>% as.character()
%>% recode(.,
                      'Deloc' = 1,
                      'Putin' = 2,
                      'Moderat' = 3,
                      'Foarte mult' = 4,
                      'Extrem de mult' = 5)}



for(i in 27:36) {
```

```
  Data[,i] =  Data[,i] %>% c() %>% unlist() %>% c() %>% as.character()
%>% recode(.,

'Foarte nesatisfacut' =1,

'Nesatisfacut' = 2,

'Nici satisfacut nici nesatisfacut' = 3,

'Satisfacut' = 4,

'Foarte satisfacut' = 5)}



Data$WHOQOL_26 = recode(Data$WHOQOL_26,
                        'Niciodata' =1,
                        'Rareori' = 2,
                        'Aproape des' = 3,
                        'Foarte des' = 4,
                        'intotdeauna' = 5
)



#### re-code HADA

for(i in 38:51) {
  Data[,i] =  Data[,i] %>% c() %>% unlist() %>% c() %>% as.character()
%>% recode(.,

'Absent' = 0,

'Usor' = 1,

'Moderat' = 2,

'Sever' = 3,

'Foarte sever' = 4)}


### re-code Cope

for(i in 52:111) {
  Data[,i] =  Data[,i] %>% c() %>% unlist() %>% c() %>% as.character()
%>% recode(.,

'De obicei nu fac asta deloc.' = 1,

'De obicei fac asta in mica masura.' = 2,

'De obicei fac asta in masura medie.' = 3,

'De obicei fac asta in mare masura.' = 4
  )}
```

```
##### total score HAMA
select(Data, starts_with('HAMA_')) %>% psych::alpha() %>% .$total
select(Data,  num_range('COPE_', c(1, 29, 38, 59)))  %>%
psych::alpha()%>% .$total

#Men_dis ## exclude scale because of reliability
#select(Data,  num_range('COPE_', c(2, 16, 31, 43))) %>% psych::alpha()

#Focus_on_ven_e ### COPE_17  excluded for alpha
select(Data,  num_range('COPE_', c(3, 28, 46))) %>% psych::alpha() %>%
.$total

#Use_ins_soci
select(Data,  num_range('COPE_', c(4, 14, 30, 45))) %>% psych::alpha()
%>% .$total

#Active_cop
select(Data,  num_range('COPE_', c(5, 25, 47, 58))) %>% psych::alpha()
%>% .$total

#Denial
select(Data,  num_range('COPE_', c(6, 27, 40, 57))) %>% psych::alpha()
%>% .$total

#Rel_coping
select(Data,  num_range('COPE_', c(7, 18, 48, 60))) %>% psych::alpha()
%>% .$total

#Humor
select(Data,  num_range('COPE_', c(8, 20, 36, 50))) %>% psych::alpha()
%>% .$total

#Beh_dise
select(Data,  num_range('COPE_', c(9, 24, 37, 51))) %>% psych::alpha()
%>% .$total

#Restraint
select(Data,  num_range('COPE_', c(10, 22, 41, 49))) %>% psych::alpha()
%>% .$total

#Use_emo
select(Data,  num_range('COPE_', c(11, 23, 34, 52))) %>% psych::alpha()
%>% .$total

#Substance_use
select(Data,  num_range('COPE_', c(12, 26, 35, 53))) %>% psych::alpha()
%>% .$total

#Acceptance
select(Data,  num_range('COPE_', c(13, 21, 44, 54))) %>% psych::alpha()
%>% .$total

#Suppression_com
select(Data,  num_range('COPE_', c(15, 33, 42, 55))) %>% psych::alpha()
%>% .$total
```

```
#Planing
select(Data,  num_range('COPE_', c(19, 32, 39, 56))) %>% psych::alpha()
%>% .$total


# physical_health drop item 3 for alpha
psych::alpha(select(Data, num_range('WHOQOL_', c(4,10,15,16,17,18))),
keys = T)$total

#psychological_health
psych::alpha(select(Data, num_range('WHOQOL_', c(5,6,7,11,19,26))), keys
= T)$total

#social_relationships
psych::alpha(select(Data, num_range('WHOQOL_', c(20, 21,22))), keys =
T)$total

#environment
psych::alpha(select(Data, num_range('WHOQOL_',
c(8,9,12,13,14,23,24,25))), keys = T)$total





Data = mutate(Data, Total_hama = rowSums(select(Data,
starts_with('HAMA_'))))

Data = mutate(Data, Pos_re = rowSums(select(Data,  num_range('COPE_',
c(1, 29, 38, 59)))))

#Data = mutate(Data, Men_dis = rowSums(select(Data,  num_range('COPE_',
c(2, 16, 31, 43)))))

Data = mutate(Data, Focus_on_ven_e = rowSums(select(Data,
num_range('COPE_', c(3, 28, 46)))))

Data = mutate(Data, Use_ins_soci = rowSums(select(Data,
num_range('COPE_', c(4, 14, 30, 45)))))

Data = mutate(Data, Active_cop = rowSums(select(Data,  num_range('COPE_',
c(5, 25, 47, 58)))))

Data = mutate(Data, Denial = rowSums(select(Data,  num_range('COPE_',
c(6, 27, 40, 57)))))

Data = mutate(Data, Rel_coping = rowSums(select(Data,  num_range('COPE_',
c(7, 18, 48, 60)))))

Data = mutate(Data, Humor = rowSums(select(Data,  num_range('COPE_', c(8,
20, 36, 50)))))

Data = mutate(Data, Beh_dise = rowSums(select(Data,  num_range('COPE_',
c(9, 24, 37, 51)))))
```

```
Data = mutate(Data, Restraint = rowSums(select(Data,  num_range('COPE_',
c(10, 22, 41, 49)))))

Data = mutate(Data, Use_emo = rowSums(select(Data,  num_range('COPE_',
c(11, 23, 34, 52)))))

Data = mutate(Data, Substance_use = rowSums(select(Data,
num_range('COPE_', c(12, 26, 35, 53)))))

Data = mutate(Data, Acceptance = rowSums(select(Data,  num_range('COPE_',
c(13, 21, 44, 54)))))

Data = mutate(Data, Suppression_com = rowSums(select(Data,
num_range('COPE_', c(15, 33, 42, 55)))))

Data = mutate(Data, Planing = rowSums(select(Data,  num_range('COPE_',
c(19, 32, 39, 56)))))

Data = mutate(Data, physical_health = (6- WHOQOL_4) +WHOQOL_10 +WHOQOL_15
+WHOQOL_16 +WHOQOL_17 +WHOQOL_18 )

Data = mutate(Data, psychological_health = WHOQOL_5 + WHOQOL_6 +WHOQOL_7
+WHOQOL_11 +WHOQOL_19 + (6 - WHOQOL_26) )

Data = mutate(Data, social_relationships = WHOQOL_20 + WHOQOL_21
+WHOQOL_22)

Data = mutate(Data, environment = WHOQOL_8 + WHOQOL_9 +WHOQOL_12 +
WHOQOL_13 + WHOQOL_14 +WHOQOL_23 +  WHOQOL_24 +WHOQOL_25)



# assumptions


# missing data percent
missing_percent = function(x){sum(is.na(x))/ length(x)*100}
apply(Data[, 111:130], 2, missing_percent)

# Univariate normality
cbind(names(Data[, 112:130]),  describe(Data[, 112:130]) %>%
as.data.frame() %>%
  select("N" = n, "M" = mean, 'SD' = sd, "Skewness" = skew, kurtosis))
%>%
  nice_table() %>% save_as_docx(path = 'Table 1.docx', align = 'left')

# Multivariate assumptions test
# Linearity assumptions test

random = rchisq(nrow(Data[, 111:130]), 14)
fake = lm(random~., data = Data[, 111:130])
standardizedResiduals = rstudent(fake)
qqnorm(standardizedResiduals)
abline(0,1)

# Normality assumption test
```

```
hist(standardizedResiduals, breaks = 44)
describe(standardizedResiduals)

#### correlation

tiff("Fig. 0.tif", family = "ArialMT", units = "cm",
     width = 50, height = 20, pointsize = 13, res = 500)
psych::cor.plot(Data[, 112:130], upper = F, stars = T)
dev.off()

### Univariante imputation

for (i in 12:130) {
  Data[,i] = as.numeric(Data[,i])

}

Database = Data[, 111:130]



### multivariate outliers


mahal = mahalanobis(Database, colMeans(Database, na.rm =T),
cov(Database))
summary(mahal)
cutoff = qchisq(.999, ncol(Database))
cutoff
summary(mahal < cutoff)
Data = subset(Data, mahal < cutoff)




### multiple regression (step wise regression)


model1 = lm(formula = Total_hama~ Pos_re +Focus_on_ven_e+Use_ins_soci+
Active_cop+Denial+Rel_coping +Humor+Beh_dise+Restraint+
             Use_emo+Substance_use+Acceptance+Suppression_com +Planing,
data = Data)


olsrr::ols_step_forward_aic(model1, details = TRUE)


########

model2 = lm(formula = physical_health ~  Pos_re
+Focus_on_ven_e+Use_ins_soci+ Active_cop+Denial+Rel_coping
+Humor+Beh_dise+Restraint+
              Use_emo+Substance_use+Acceptance+Suppression_com +Planing,
data = Data)


olsrr::ols_step_forward_aic(model2, details = TRUE)
```

```
#######

model3 = lm(formula = psychological_health ~  Pos_re
+Focus_on_ven_e+Use_ins_soci+ Active_cop+Denial+Rel_coping
+Humor+Beh_dise+Restraint+
              Use_emo+Substance_use+Acceptance+Suppression_com +Planing,
data = Data)


olsrr::ols_step_forward_aic(model3, details = TRUE)



#######

model4 = lm(formula = social_relationships~  Pos_re
+Focus_on_ven_e+Use_ins_soci+ Active_cop+Denial+Rel_coping
+Humor+Beh_dise+Restraint+
              Use_emo+Substance_use+Acceptance+Suppression_com +Planing,
data = Data)


olsrr::ols_step_forward_aic(model4, details = TRUE)


#######

model5 = lm(formula = environment ~  Pos_re
+Focus_on_ven_e+Use_ins_soci+ Active_cop+Denial+Rel_coping
+Humor+Beh_dise+Restraint+
              Use_emo+Substance_use+Acceptance+Suppression_com +Planing,
data = Data)


olsrr::ols_step_forward_aic(model5, details = TRUE)
```
