# Supplementary material for: Coping Mechanisms during the War in Ukraine: A Cross-Sectional Assessment among Romanian Population
Source: Healthcare (Basel). 2023 May 13;11(10):1412. doi: 10.3390/healthcare11101412 (PMC10218351; doi:10.3390/healthcare11101412)
Supplement: Supplementary file 1 [file healthcare-11-01412-s001.zip › Table S1 - Stepwise regression.pdf]

Table S1

The statistics for the stepwise regression.

	b	SE	β	t	Sig.	CI lower	CI upper	AIC	R^2
**Model 1 Anxiety symptoms (DV)**									
Focusing on and venting emotions	1.41	0.11	0.30	13.40	.000	1.20	1.62	11226.50	0.14
Behavioral disengagement	0.88	0.11	0.20	8.28	.000	0.67	1.09	11115.69	0.20
Acceptance	-0.39	0.10	-0.10	-3.89	.000	-0.58	-0.19	11074.87	0.22
Substance use	0.71	0.10	0.16	6.82	.000	0.51	0.91	11035.07	0.24
Humor	-0.26	0.07	-0.09	-3.74	.000	-0.39	-0.12	11015.43	0.25
Positive reinterpretation and growth	-0.30	0.12	-0.07	-2.57	.010	-0.53	-0.07	11012.24	0.26
Restraint	0.20	0.11	0.05	1.85	.064	-0.01	0.41	11010.79	0.26
**Model 2 Physical health (DV)**									
Behavioral disengagement	-0.37	0.05	-0.22	-8.11	.000	-0.46	-0.28	8467.51	0.09
Focusing on and venting emotions	-0.45	0.04	-0.24	-10.38	.000	-0.53	-0.36	8368.49	0.15
Positive reinterpretation and growth	0.33	0.05	0.19	6.85	.000	0.23	0.42	8256.05	0.21
Substance use	-0.21	0.04	-0.11	-4.90	.000	-0.29	-0.13	8239.36	0.22
Humor	0.07	0.03	0.06	2.42	.016	0.01	0.13	8230.92	0.22
Denial	0.14	0.05	0.07	2.72	.007	0.04	0.23	8227.74	0.23
Acceptance	0.12	0.04	0.08	2.85	.004	0.04	0.20	8223.32	0.23
Restraint	-0.08	0.04	-0.05	-1.85	.064	-0.17	0.01	8221.88	0.23
**Model 3 Psychological health (DV)**									
Positive reinterpretation and growth	0.38	0.05	0.22	8.19	.000	0.29	0.47	8369.52	0.12
Focusing on and venting emotions	-0.51	0.05	-0.28	-11.42	.000	-0.60	-0.42	8189.71	0.22
Behavioral disengagement	-0.42	0.04	-0.25	-9.75	.000	-0.50	-0.34	8081.89	0.27
Substance use	-0.26	0.04	-0.15	-6.54	.000	-0.34	-0.18	8047.11	0.29
Humor	0.09	0.03	0.08	3.24	.001	0.03	0.14	8033.18	0.30
Use of emotional social support	0.11	0.03	0.09	3.54	.000	0.05	0.17	8022.89	0.30
Denial	0.15	0.05	0.08	3.16	.002	0.06	0.24	8015.00	0.31
Acceptance	0.10	0.04	0.07	2.58	.010	0.02	0.17	8012.19	0.31
Restraint	-0.09	0.04	-0.05	-2.19	.028	-0.17	-0.01	8009.68	0.31
Religious coping	0.04	0.02	0.04	1.95	.052	0.00	0.08	8007.87	0.31

**Model 4 Social relationships**									
Positive reinterpretation and growth	0.12	0.03	0.12	4.42	.000	0.07	0.17	6918.96	0.07
Behavioral disengagement	-0.18	0.03	-0.18	-7.09	.000	-0.23	-0.13	6853.12	0.11
Use of emotional social support	0.20	0.02	0.27	9.94	.000	0.16	0.24	6813.34	0.13
Focusing on and venting emotions	-0.24	0.03	-0.22	-8.15	.000	-0.30	-0.18	6739.56	0.17
Humor	0.06	0.02	0.09	3.57	.000	0.03	0.10	6730.76	0.18
Substance use	-0.07	0.03	-0.07	-2.73	.007	-0.12	-0.02	6725.32	0.18
**Model 5 Environment**									
Positive reinterpretation and growth	0.28	0.05	0.16	5.45	.000	0.18	0.39	8704.58	0.06
Behavioral disengagement	-0.24	0.045	-0.13	-4.91	.000	-0.34	-0.15	8637.54	0.10
Focusing on and venting emotions	-0.41	0.05	-0.21	-7.70	.000	-0.51	-0.30	8599.94	0.12
Use of emotional social support	0.21	0.04	0.16	5.87	.000	0.14	0.28	8567.35	0.14
Substance use	-0.16	0.05	-0.09	-3.46	.001	-0.26	-0.07	8558.94	0.15
Religious coping	-0.07	0.03	-0.07	-2.72	.007	-0.12	-0.02	8551.41	0.15
Acceptance	0.10	0.04	0.06	2.34	.019	0.02	0.19	8546.73	0.16
Denial	-0.10	0.06	-0.05	-1.75	.080	-0.21	0.01	8545.64	0.16

Note: β – standardized regression coefficient; b – unstandardized regression coefficient; SE – Standard Error of b; sig. – p values corresponding to the estimates, AIC - Akaike's Information Criteria; CI – 95% confidence interval for b; DV – dependent variable; R^2 – represents the variance explained in the dependent variables as predictors are added one by one.
